# Towards generalisable foundation models for brain MRI

**DOI:** 10.1038/s44303-026-00176-5

**Published:** 2026-05-29

**Authors:** Moona Mazher, Geoff J. M. Parker, Daniel C. Alexander

**Affiliations:** 1 UCL Hawkes Institute, Department of Computer Science, University College London, London, UK; 2 Department of Computer Science, University College London, London, UK; 3 Department of Medical Physics and Biomedical Engineering, University College London, London, UK; 4 Bioxydyn Limited, Manchester, UK

**Keywords:** Computational biology and bioinformatics, Engineering, Health care, Mathematics and computing, Medical research, Neuroscience

## Abstract

Foundation models trained with self-supervised learning are increasingly enabling scalable and generalisable solutions in medical imaging. However, most existing foundation models are designed for 2D natural images and do not explicitly leverage the structure of brain MRI data. We introduce BrainFound, a self-supervised foundation model for brain MRI that adopts a slice-based learning strategy, processing MRI volumes as sequences of 2D slices. This approach enables efficient learning while capturing contextual information across slices. The framework supports both single-modality and multimodal inputs (e.g., T1, T2, FLAIR), allowing integration of complementary structural information. We evaluate BrainFound across multiple downstream tasks, including neurodegenerative disease detection, tumour grading, and brain tissue segmentation, using diverse public datasets. The model consistently outperforms both supervised and self-supervised baselines, particularly in label-scarce and cross-dataset settings, demonstrating strong generalisation. These results highlight the effectiveness of slice-based self-supervised learning as a scalable approach for brain MRI analysis. BrainFound provides a flexible foundation for neuroimaging applications with potential for both clinical deployment and large-scale research.

## Introduction

Radiological imaging is central to modern medicine, offering detailed, non-invasive insights for diagnosis and treatment planning^1,2^. With rising imaging use and a shortage of radiologists, the clinical workload has become unsustainable, requiring radiologists to interpret one image every 3–4 s to keep up^3,4^. This burden leads to delays and diagnostic errors^5,6^, highlighting the need for automated tools to enhance accuracy and efficiency.

Deep learning has shown promise in radiology, but its success depends on large, labelled datasets, which are expensive and time-consuming to obtain^7,8^. Additionally, supervised models often struggle to generalise across domains and data shifts^9^. Self-supervised learning (SSL) addresses these limitations by learning from unlabelled data, enabling scalable and robust model development^10^.

Foundation Models (FMs), trained using SSL on large-scale data, have achieved strong generalisation and few-shot performance across a diverse range of tasks^11–13^. However, most current models are specifically designed to deal with 2D natural imaging datasets, while medical imaging datasets require 3D models to handle volumetric MRIs^14^, which are crucial to avoid the loss of spatial context critical for accurate diagnosis.

3D models can capture spatial dependencies across slices, which is essential for tasks such as lesion detection in conditions like multiple sclerosis or neuro-oncology, as well as for assessing brain atrophy in dementia. Working directly with 3D data helps preserve anatomical details. Although the majority of existing machine learning models operate on single-contrast MRI data, our use of multimodal/multi-contrast inputs (e.g., T1, T2, FLAIR) improves performance across a range of tasks, including disease detection and segmentation, by capturing complementary structural information^15,16^.

Self-supervised learning (SSL) has shown promise in brain-related tasks such as Alzheimer’s disease detection, tumour classification, post-traumatic stress disorder (PTSD) prediction, and brain age estimation^17–19^. However, many existing efforts focus on specific applications, often lacking broader generalisation. Furthermore, although the comparative strengths of contrastive and generative SSL approaches are well studied in natural image domains^20–22^, their relative advantages in medical imaging remain underexplored. Additionally, challenges related to interpretability and effective integration of multimodal inputs in SSL models continue to limit their clinical utility in neuroimaging.

However, training true 3D models from scratch is computationally expensive and requires large-scale data, motivating the need for efficient alternatives that can approximate 3D context without full volumetric processing.

To address these challenges, we propose BrainFound, a slice-based self-supervised foundation model tailored for brain MRI. Unlike true 3D architectures, BrainFound processes 2D slices sequentially to approximate anatomical context across depth, balancing computational efficiency with spatial awareness. Built by extending the 2D DINOv2^23^ framework to brain MRI via a slice-wise strategy, BrainFound approximates long-range anatomical context and captures detailed brain structures crucial for accurate analysis.

BrainFound demonstrates strong performance across tasks, effectively handling both diffuse neurodegenerative changes and focal pathologies, such as tumours. By combining general visual priors with domain-adapted SSL, BrainFound establishes a strong benchmark for SSL in brain imaging. We comprehensively evaluate BrainFound across classification, segmentation, cross-dataset generalisation, and few-shot learning tasks spanning neurodegenerative, oncological, and fetal brain imaging.

Our key contributions are:

1. We designed BrainFound, a slice-based self-supervised model adapted from DINOv2^23^ for brain MRI representation learning using sequential 2D MRI slices for transferable neuroimaging analysis.
2. Our model can process both single- and multimodal MRI inputs (in our current implementation, T1, T2, FLAIR, but adaptable to alternative/more contrasts), thereby enhancing robustness and enabling versatile diagnostic applications.
3. We demonstrate consistent performance across varying voxel resolutions, including both isotropic (e.g., 1-2 mm^3^) and anisotropic voxel sizes, which improves generalisability and practical applicability across diverse clinical imaging protocols.
4. We develop a multitask SSL framework capable of supporting a broad spectrum of brain MRI tasks, including multi-disease classification (e.g., multiple dementia types and tumour grading) and multi-region anatomical and tumour segmentation, demonstrating consistent performance across both diagnostic and structural analyses.

## Results

We evaluate BrainFound on two core neuroimaging tasks: disease classification and brain structure/pathology segmentation. The BrainFound performance is compared against both supervised and self-supervised baselines across multiple public datasets and modalities (T1, T2, FLAIR).

For disease detection, we benchmark BrainFound against SL_ImageNet (supervised ImageNet pretraining), SSL_DINOv2 (self-supervised on LVD-142M)^23^, SSL_BrainImages (self-supervised on brain MRI data), and Med3D_ResNet50, a 3D ResNet model pretrained on a heterogeneous set of volumetric medical images^24^. Med3D processes full 3D volumes, providing a reference for performance achievable with true volumetric representations. In contrast, BrainFound operates on slice-wise 2D approximations of MRI volumes; despite the potential loss of information along the third dimension, BrainFound still outperforms Med3D_ResNet50. Additional comparisons include SimCLR^20^, SwAV^25^, MoCo-v3^21^, and Masked Autoencoder^22^ to assess the benefits of the DINOv2-based self-distillation approach.

For segmentation, we evaluate BrainFound against strong baselines such as U-Net and 3D state-of-the-art models, including MedSAM^26^, Hi-End-MAE^27^, SSL_DINOv2, and SSL_BrainImages. Evaluations were conducted across multiple datasets using Dice and HD95 as standard metrics. Detailed results and comparisons for both tasks are presented below.

### Disease detection task

We first evaluated whether BrainFound learns robust and transferable representations across heterogeneous disease detection tasks, including both neurodegenerative disorders and brain tumour grading. These experiments assess the ability of the pretrained framework to capture disease-relevant anatomical patterns across distinct pathologies, datasets, and imaging distributions.

**Fig. 1:**
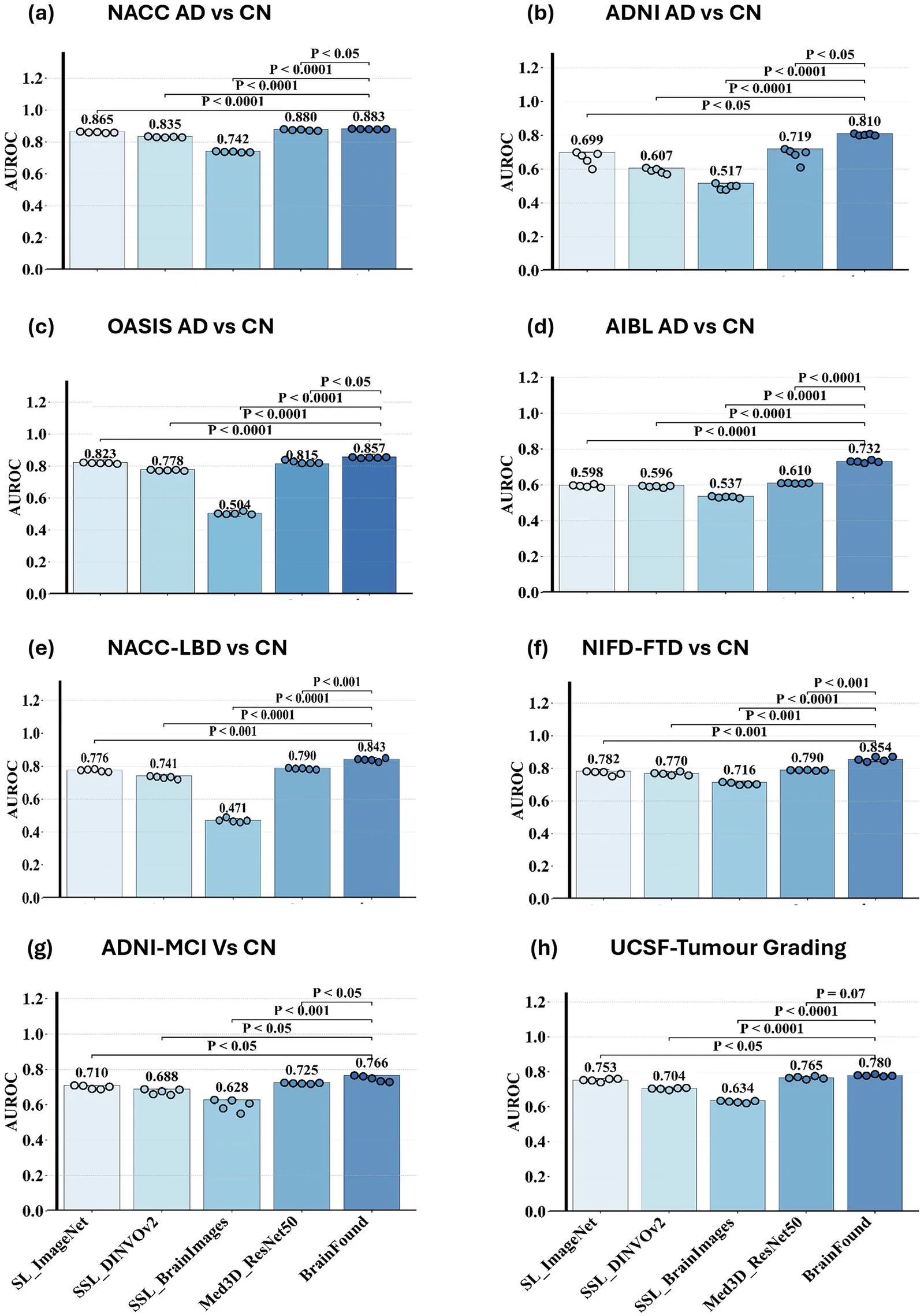
Comparative evaluation of pretrained representation learning strategies across multiple downstream disease detection tasks. AUROC performance comparison across pretraining strategies for Alzheimer’s disease, Lewy body dementia, frontotemporal dementia, mild cognitive impairment detection, and multiclass tumour grading. **a**–**d** show Alzheimer’s disease detection on the NACC, ADNI, OASIS and AIBL cohorts, respectively; **e** shows Lewy body dementia detection (NACC); **f** shows frontotemporal dementia detection (NIFD); **g** shows mild cognitive impairment detection (ADNI); and **h** shows glioma tumour grading on the UCSF dataset. Models were initialised with one of five pretraining methods: SL_ImageNet, SSL_DINOv2, SSL_BrainImages, Med3D_ResNet50, or the proposed BrainFound. Each task was evaluated using 5-fold cross-validation. The mean AUROC across folds is shown as the height of each bar. Individual fold results are indicated by the five dots on each bar, illustrating variability across folds. Statistically significant improvements (*p* < 0.05, two-sided *t*-test^31^) are indicated above the bars.

Alzheimer’s Disease Detection: BrainFound was evaluated across four independent cohorts (NACC, ADNI, OASIS, and AIBL) to assess robustness across heterogeneous clinical populations. The model discriminated Alzheimer’s disease (AD) patients from cognitively normal controls using internal cross-validation within each dataset, as given in Fig. 1a–d.

On the NACC cohort, BrainFound achieved an AUROC of 0.883, outperforming Med3D_ResNet50 (0.880), SL_ImageNet (0.865), SSL_DINOv2 (0.835), and SSL_BrainImages (0.742). Similar trends were observed across external cohorts. On ADNI, AUROC values were 0.810 (BrainFound), 0.719 (Med3D_ResNet50), 0.699 (SL_ImageNet), 0.607 (SSL_DINOv2), and 0.517 (SSL_BrainImages). On OASIS, performance reached 0.857 compared with 0.815, 0.823, 0.778, and 0.504, respectively. On AIBL, AUROC scores were 0.732, 0.610, 0.598, 0.596, and 0.537.

Across all four cohorts, BrainFound consistently achieved the highest performance, including improvements over Med3D_ResNet50, a 3D CNN pretrained on large-scale medical imaging data. While Med3D provides domain-specific initialisation, the dual-stage strategy employed by BrainFound—combining large-scale self-supervised pretraining on natural images (DINOv2) with subsequent domain adaptation on multimodal brain MRI—yields more transferable and disease-sensitive representations. These results highlight the advantage of integrating large-scale representation learning with domain adaptation rather than relying solely on medical-domain pretraining.

Lewy Body Dementia Detection: On the NACC dataset, BrainFound achieved an AUROC of 0.843 for LBD detection, with Med3D_ResNet50 (0.790), SL_ImageNet at 0.776, SSL_DINOv2 at 0.741, and SSL_BrainImages at 0.471 (Fig. 1). This indicates the model captures disease-specific structural patterns beyond Alzheimer’s disease.

Frontotemporal Dementia Detection: For FTD on the NIFD dataset, AUROC scores were 0.854 (BrainFound), 0.790 (Med3D_ResNet50), 0.782 (SL_ImageNet), 0.770 (SSL_DINOv2), and 0.716 (SSL_BrainImages) (Fig. 1).

Mild Cognitive Impairment Detection: On the ADNI cohort, AUROC scores for MCI detection were 0.766, 0.725, 0.698, 0.615, and 0.528 for BrainFound, Med3D_ResNet50, SL_ImageNet, SSL_DINOv2, and SSL_BrainImages, respectively (Fig. 1).

Brain Tumour Grading (Oncological Disease Detection): In the UCSF glioma grading task, BrainFound achieved an AUROC of 0.780, compared with 0.765 (Med3D_ResNet50), 0.753 (SL_ImageNet), 0.704 (SSL_DINOv2), and 0.634 (SSL_BrainImages) (Fig. 1). Lower AUROC for SSL_BrainImages reflects limitations of self-supervised learning trained from scratch on brain MRI. The combination of pretrained DINOv2 weights with domain adaptation enables more informative and transferable representations.

### Cross-dataset external evaluation

Cross-dataset external validation is critical in neuroimaging because models often fail under domain shift caused by scanner variability, acquisition protocols, and cohort differences. We therefore evaluated cross-dataset transfer to assess whether slice-wise SSL representations remain robust across independent populations.

**Fig. 2:**
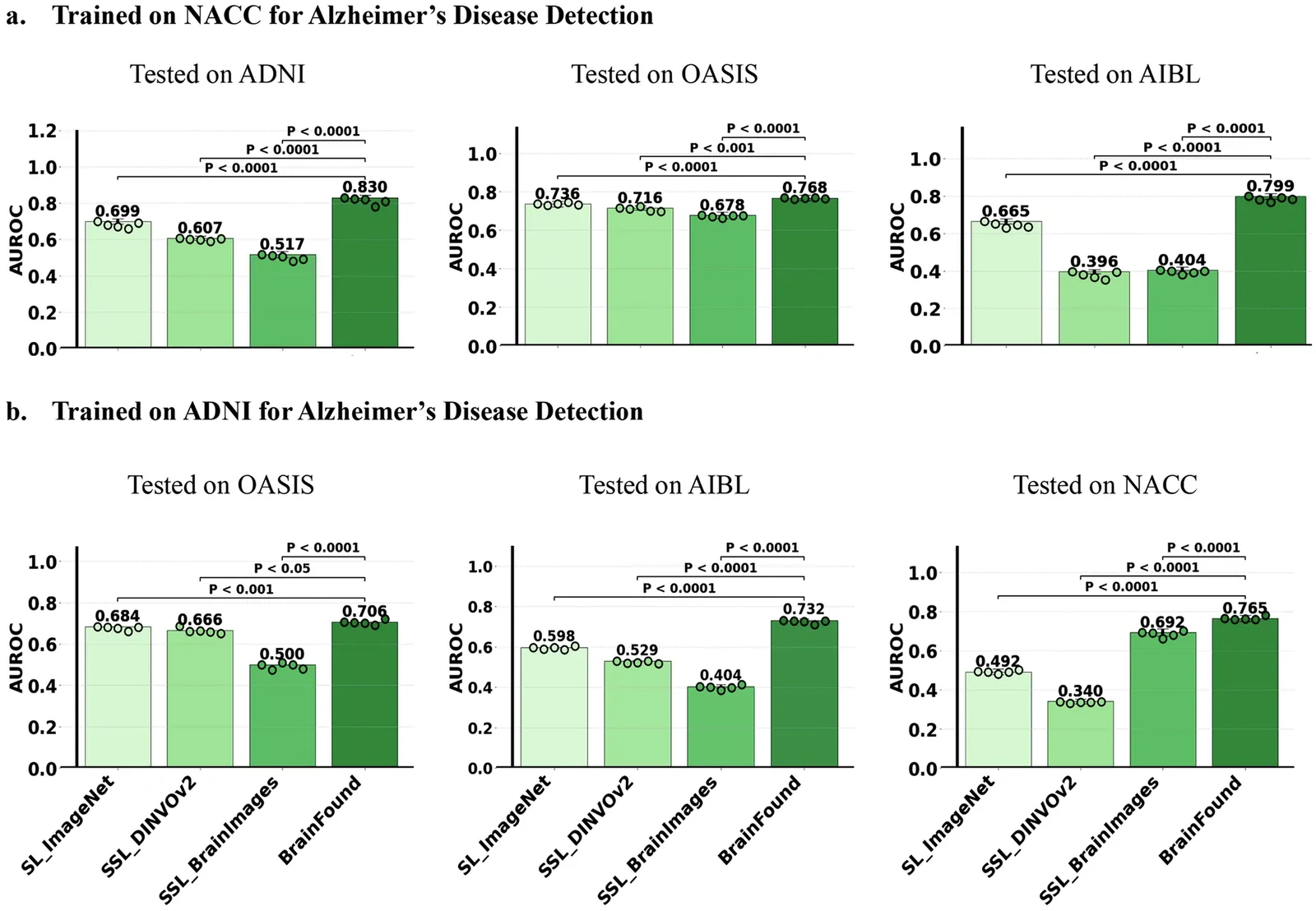
Cross-data external evaluation of pretraining strategies. Comparison of AUROC performance across different pretraining strategies under an external evaluation setting, where models are pretrained on one dataset and evaluated on a distinct, unseen dataset. **a** shows models trained on the NACC cohort and externally evaluated on the ADNI, OASIS and AIBL cohorts. **b** shows models trained on the ADNI cohort and externally evaluated on the NACC, OASIS and AIBL cohorts. Each panel shows AUROC scores for SL_ImageNet, SSL_DINOv2, SSL_BrainImages, and BrainFound. Statistical significance (*p* < 0.05, two-sided *t*-test) is indicated above the bars. Dots represent AUROC values from five-fold evaluation splits.

Six cross-cohort evaluations for AD classification tested models on independent cohorts. When trained on NACC, BrainFound achieved AUROCs of 0.830 (ADNI), 0.880 (OASIS), and 0.799 (AIBL). When trained on ADNI, AUROCs were 0.765, 0.706, and 0.732, respectively (Fig. 2). While SSL_BrainImages shows relatively strong performance in this specific ADNI → NACC transfer, this is an exception; in all other tasks, it remains the lowest-performing baseline. BrainFound benefits from large-scale natural-image pretraining (DINOv2, 142M images) combined with MRI-specific self-supervision, resulting in consistently superior cross-domain performance. This contextualises the observed variability and reinforces the value of combining natural-image priors with domain-specific adaptation.

### Comparison of state-of-the-art self-supervised learning strategies

Different SSL paradigms capture complementary visual representations, but their relative effectiveness in brain MRI remains unclear. We therefore compared self-distillation, contrastive, clustering-based, and reconstruction-based SSL approaches under identical experimental settings.

**Fig. 3:**
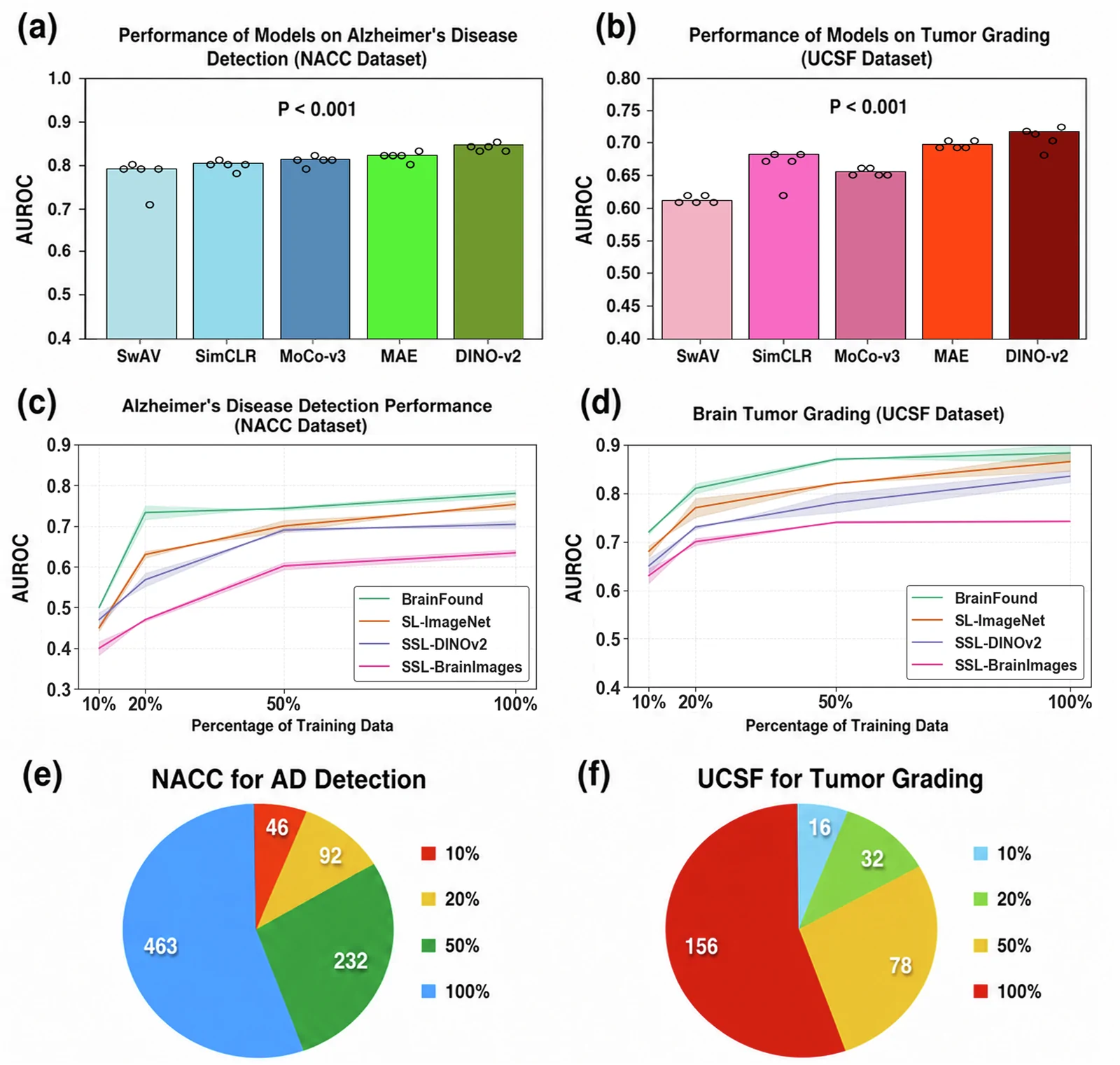
Comparison of foundation models and few-shot learning. **a** Performance comparison of state-of-the-art foundation models with DINOv2 for Alzheimer’s disease detection on the NACC dataset. **b** Performance comparison of state-of-the-art foundation models with DINOv2 for tumour grading on the UCSF dataset. **c**, **d** Few-shot learning comparisons of the baseline models with BrainFound for Alzheimer’s disease detection and tumour grading. **e**, **f** Data distribution for few-shot learning.

We compared DINOv2 with SwAV, SimCLR, MoCo-v3, and MAE for AD detection (NACC) and tumour grading (UCSF) using multimodal MRI (Fig. 3a, b). For AD, DINOv2 achieved the highest AUROC, followed by MAE, with SwAV, SimCLR, and MoCo-v3 lower. For tumour grading, DINOv2 and MAE were top-performing, with SimCLR slightly outperforming MoCo-v3.

### Evaluation of few-shot learning

Annotated neuroimaging datasets are often limited due to the expertise and cost required for labelling. We therefore evaluated performance under few-shot settings to determine whether pretrained slice-wise representations improve label efficiency and maintain robustness with limited supervision.

Few-shot experiments used 10%, 20%, 50%, and 100% of labelled training data (Fig. 3c, d). BrainFound maintained a higher AUROC even under severe data scarcity. Figure 3e, f visualises the absolute and relative number of labelled samples, with 95% confidence intervals.

### Brain MRI segmentation

To evaluate whether the learned representations transfer beyond classification tasks, we assessed anatomical and pathological segmentation performance on fetal brain tissue segmentation and brain tumour subtype segmentation datasets.

**Fig. 4:**
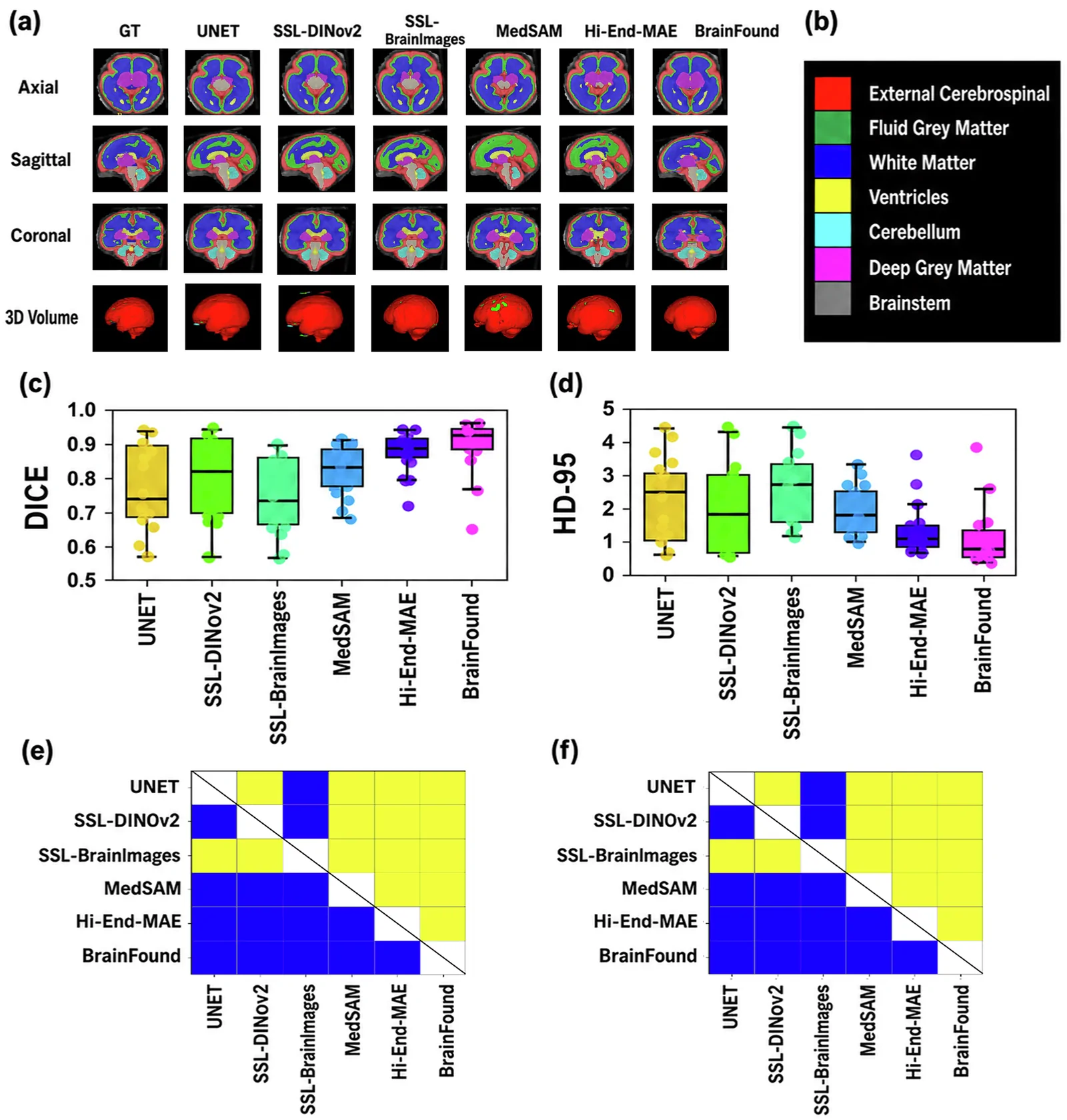
Fetal brain tissue segmentation results. Fetal brain tissue segmentation results from the FeTA dataset. **a** shows the visualisation of different views of the brain (top-down) for ground-truth (GT), and BrainFound predicted masks, along with the prediction of other baseline models, with coloured regions representing different brain structures. **c**, **d** Boxplots of the Dice coefficient and HD 95 comparing BrainFound with other baseline segmentation methods for the fetal brain tissue segmentation dataset. **e**, **f** Pairwise comparison matrix of model performance for Dice (higher is better) and HD95 (lower is better). Blue indicates the model performs better than the compared model (high rank), yellow indicates worse performance (low rank), and white marks the diagonal (self-comparison). **b** shows the class-mapping function for the axial, sagittal, and coronal slices.

Fetal Brain Tissue Segmentation (FeTA 2021): BrainFound was trained on 80 T2-weighted fetal MRI scans labelled into seven tissue types^28^. Dice and HD95 metrics indicate higher volumetric accuracy and boundary precision compared with baselines (Fig. 4c–f). Visualisations of predicted versus ground-truth masks are shown in Fig. 4a.

**Fig. 5:**
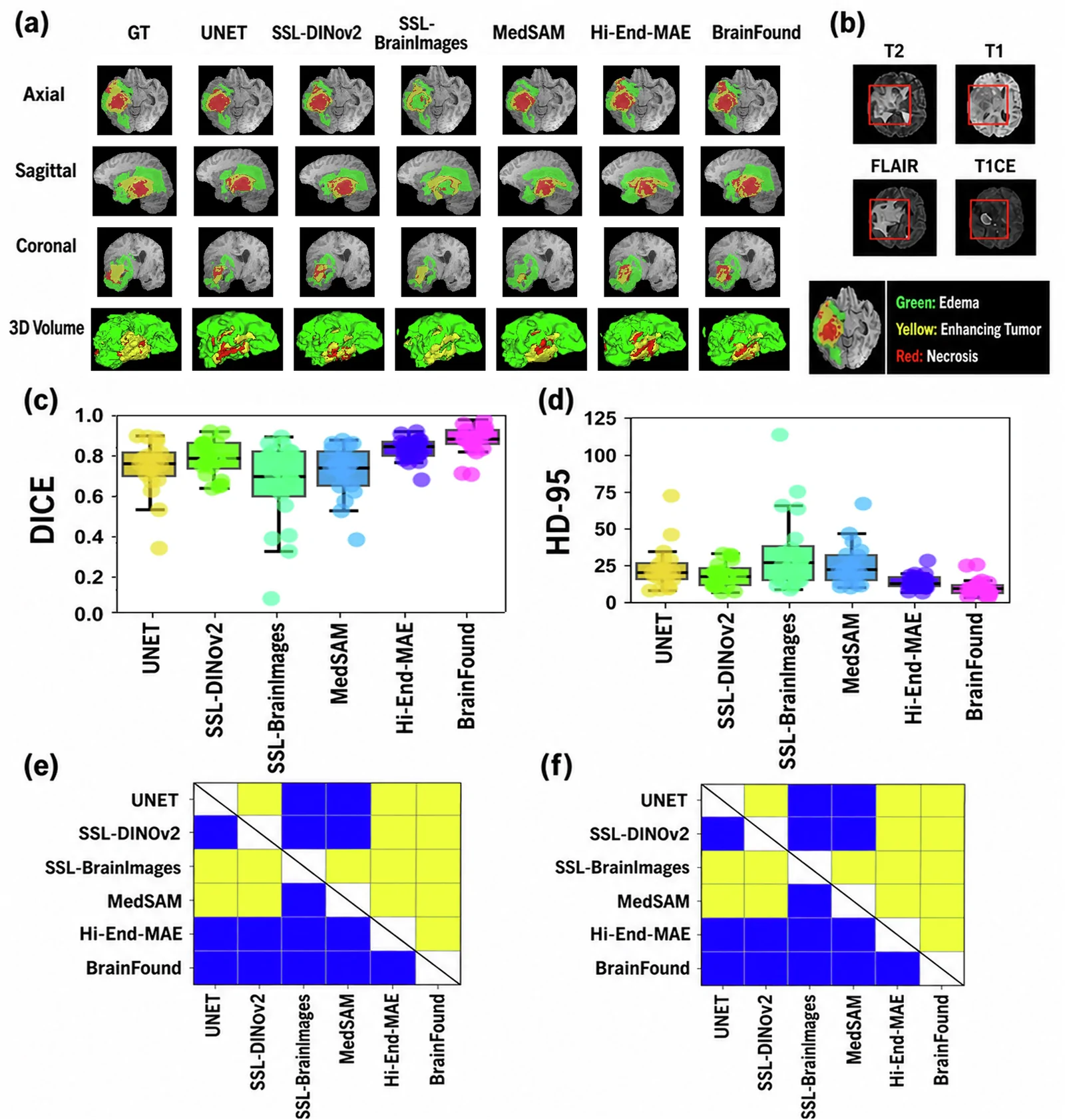
Brain tumour subtype segmentation results. Brain tumour subtype segmentation results from the BraTS dataset. Image (**a**) shows the visualisations of the different views of the brain (top-down) for ground-truth (GT) and BrainFound predicted masks, along with the prediction of other baseline models, with coloured regions representing different brain tumour labels. **c**, **d** Boxplots of the Dice coefficient and HD 95 comparing BrainFound with other baseline segmentation methods for the brain tumour subtypes segmentation dataset. **e**, **f** Pairwise comparison matrix of model performance for Dice (higher is better) and HD95 (lower is better). Blue indicates the model performs better than the compared model (high rank), yellow indicates worse performance (low rank), and white marks the diagonal (self-comparison). **b** shows T1, T2, T1CE, and FLAIR MRI sequences for a patient with brain tumours are highlighted in red boxes.

Brain Tumour Subtype Segmentation (BraTS 2020): BrainFound segmented glioma subregions (enhancing tumour, oedema, necrotic core) using multi-contrast MRI (T1, T2, FLAIR). Dice and HD95 metrics indicate consistent volumetric accuracy and boundary precision (Fig. 5c–f). Visual comparisons are in Fig. 5a, with T1, T2, T1CE, and FLAIR sequences highlighted in Fig. 5b^27^.

## Discussion

This study introduces BrainFound, a self-supervised foundation model designed to learn generalisable representations from brain MRI across multiple downstream tasks, including disease detection and image segmentation. BrainFound adopts a slice-based self-supervised learning strategy in which MRI volumes are decomposed into axial slices and processed sequentially using a 2D Vision Transformer backbone. Building on the self-distillation paradigm of DINOv2, the framework combines large-scale natural-image pretraining with domain-specific self-supervised adaptation on brain MRI data. This strategy enables BrainFound to retain transferable visual priors while adapting to domain-specific anatomical structures, providing a practical balance between representation quality, scalability, and computational efficiency.

Previous advances in medical imaging have demonstrated the effectiveness of transfer learning from natural-image datasets such as ImageNet for downstream radiology applications^29,30^. More recently, self-supervised learning approaches, including SimCLR, BYOL, MAE, and DINOv2, have shown that large-scale unlabelled data can produce robust and transferable visual representations. In medical imaging, SSL foundation models such as RETFound^31^, 3D-Heart_Seg^32^, and DINOv2-based approaches^33^ further highlight the potential of self-supervised learning to improve generalisation and reduce dependence on annotated datasets.

BrainFound builds upon this paradigm by adapting DINOv2 representations to brain MRI through a slice-wise training strategy. By initialising from DINOv2 pretrained weights and subsequently performing self-supervised training on approximately 10,000 unlabelled MRI volumes, the model benefits from both general visual priors and domain-specific anatomical adaptation. This hybrid pretraining strategy enables BrainFound to learn representations that generalise effectively across heterogeneous disease detection tasks, independent cohorts, and limited-supervision settings without relying on extensive labelled data. The framework also supports both single- and multimodal MRI sequences, including T1, T2, FLAIR, and contrast-enhanced imaging, enabling integration of complementary anatomical information across modalities and improving diagnostic robustness.

Comprehensive benchmarking demonstrated that BrainFound consistently outperformed representative supervised and self-supervised baselines across neurodegenerative disease detection, tumour grading, and segmentation tasks. Comparisons with SL_ImageNet, SSL_DINOv2, SSL_BrainImages, and Med3D_ResNet50^24^ suggest that combining large-scale natural-image pretraining with MRI-specific adaptation produces more stable and transferable representations than either supervised transfer learning or MRI-only self-supervised training from scratch. Importantly, BrainFound also demonstrated strong robustness under domain shift and limited supervision. External evaluation across independent cohorts showed that the learned representations generalise across variations in scanner protocols, acquisition settings, and patient populations, while few-shot experiments demonstrated that pretrained representations maintain strong performance even with limited labelled data. Additional comparisons with SimCLR, MAE, MoCo v3, SwAV, and Hi-End-MAE further indicate that self-distillation-based representation learning provides robust and discriminative features for heterogeneous brain MRI analysis.

Notably, BrainFound also demonstrated strong transferability to segmentation tasks, outperforming widely used architectures such as UNet and multiple self-supervised baselines. The framework achieved competitive or superior Dice and HD95 scores relative to advanced volumetric approaches such as MedSAM and Hi-End-MAE despite operating on 2D slices rather than explicitly modelling full three-dimensional spatial context. These findings suggest that slice-based representation learning can effectively leverage sequential anatomical slice information while maintaining computational efficiency and compatibility with scalable pretrained 2D transformer architectures.

Several limitations should be acknowledged. Processing brain MRI as ordered 2D slices allows efficient use of pretrained 2D transformers, but does not explicitly model full 3D spatial dependencies. Additionally, although independent test datasets were excluded from model development, downstream training datasets were incorporated into the self-supervised pretraining stage due to the limited availability of large-scale public brain MRI data, which may slightly inflate development-phase performance estimates. Future work will focus on extending this framework toward fully volumetric self-supervised architectures capable of directly modelling three-dimensional anatomical relationships. Expanding the scale and diversity of pretraining datasets across institutions, scanners, and populations will also be important for improving robustness and generalisation. Furthermore, BrainFound could be extended to additional MRI modalities, including susceptibility-weighted imaging, diffusion imaging, and functional MRI, and integrated with multimodal foundation models combining imaging and clinical text to enhance downstream clinical utility.

Overall, BrainFound demonstrates that slice-based self-supervised learning can effectively bridge the gap between large-scale natural-image representation learning and domain-specific medical imaging tasks. By combining strong pretrained visual representations with large-scale MRI adaptation, the proposed framework achieves robust performance across disease detection, segmentation, cross-dataset evaluation, and few-shot learning while maintaining practical computational efficiency. These findings suggest that BrainFound provides a promising foundation for scalable neuroimaging research and future clinical applications in brain MRI analysis.

## Methods

We developed BrainFound, a self-supervised learning (SSL) foundation model for single-modality and multimodal brain MRI. Trained on large-scale unlabelled data, BrainFound learns generalisable representations that support diverse downstream tasks, including disease detection and anatomical or pathological segmentation. The model is designed to operate with either single-modality or combined-modality inputs, allowing flexible use across heterogeneous datasets. The following subsections provide details on datasets, SSL architecture, baseline comparisons, training strategy, and downstream evaluation.

### Datasets

For BrainFound, we curated ~ 10,000 volumetric brain MRI images across three modalities (T1, T2, FLAIR) from twelve public datasets: BraTS^34^, ReMIND^35^, UPENN-GBM^36^, UCSF-PDGM^37^, Burdenko-GBM-Progression^38^, HABS^39^, OASIS^40^, ADNI^41^, NACC^42^, AIBL^43^, NIFD^44^, and FeTA^28^ (Supplementary Fig. 1).

This study used only publicly available datasets containing anonymised human data. No new data were collected from human participants. Therefore, ethical approval and informed consent were not required. All procedures performed in this study were conducted in accordance with the principles of the Declaration of Helsinki.

For datasets used in both self-supervised pretraining and downstream tasks, 15% of volumes were held out as a test set before any training began from each dataset. The remaining 85% was used for SSL pretraining. For downstream fine-tuning, we split this 85% data into five training/validation folds: in each fold, models were trained on the training split and validated on the validation split. After training and validation across all five folds, each of the five models was evaluated on the strictly held-out 15% test set. The final metrics (AUROC, Dice, HD95) are averaged across the five models. Further details on datasets are provided in Supplementary Table 1.

Only T1-, T2-, and FLAIR-weighted images with complete volumetric coverage were included; images with severe artifacts, motion blur, or incomplete acquisition were excluded. Subjects spanned a wide age range ( ~ 18-90 years). Sex and diagnostic class distributions were balanced wherever possible, using random sampling or stratified selection for uneven cohorts.

**Fig. 6:**
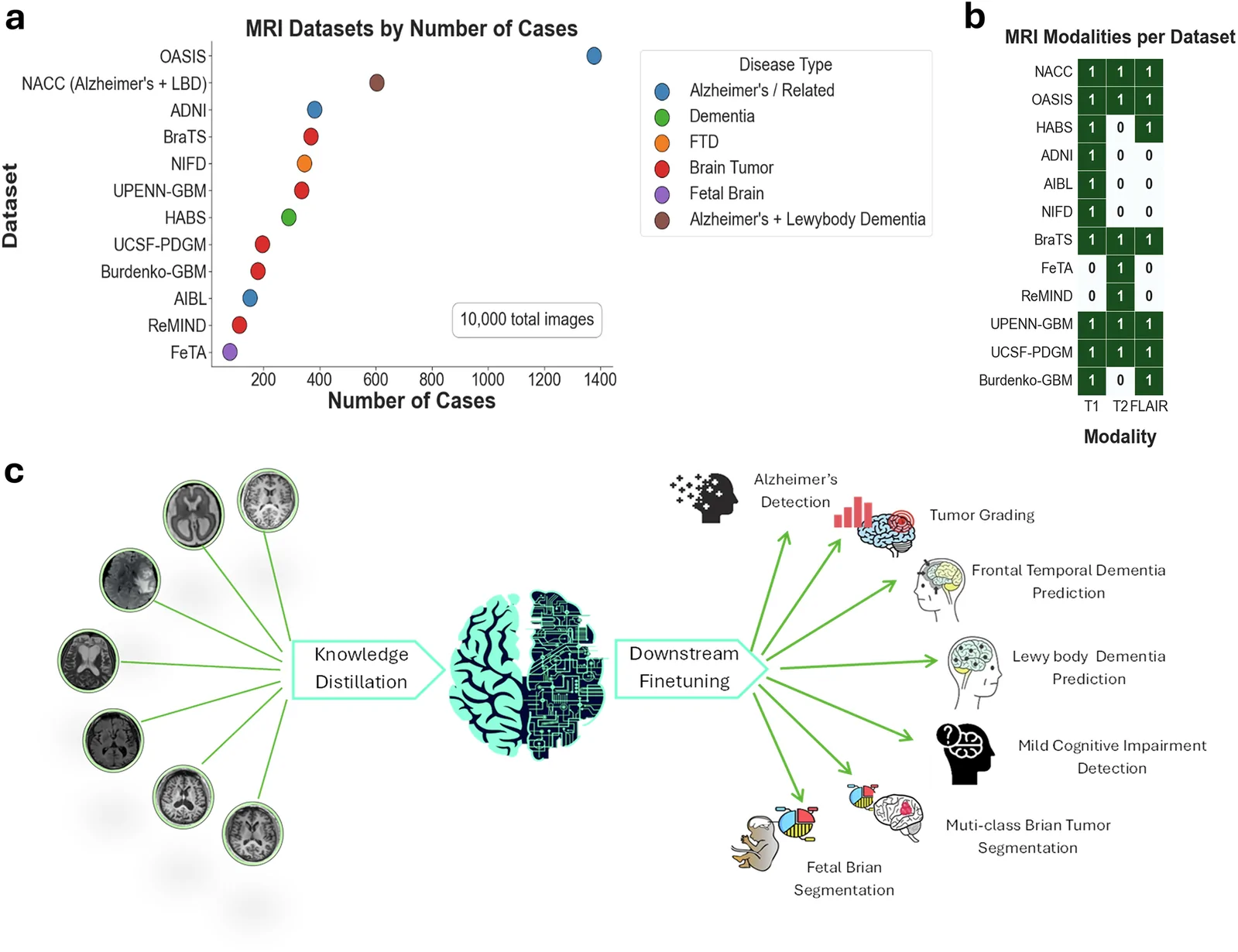
MRI dataset overview for SSL and downstream tasks. Overview of MRI datasets and modalities, for the SSL and downstream workflow. **a** Number of MRI cases per dataset, colour-coded by disease type, including Alzheimer’s disease, frontotemporal dementia (FTD), brain tumours, and fetal brain conditions. **b** Imaging modality availability (T1, T2, FLAIR) for each dataset, indicating multimodal coverage relevant for pre-training. **c** Overall framework: multiple datasets and modalities are used in a knowledge distillation-based SSL pretraining stage, followed by downstream fine-tuning showcasing generalisation across disease detection and image segmentation tasks.

Our framework accommodates both single- and multi-modality scans. The details of scan types, dataset usage, and modality coverage across all datasets are summarised in Fig. 6a–c. An example of single-versus combined-modality performance on Alzheimer’s disease detection in the NACC dataset is provided in Supplementary Fig. 4, illustrating that combining T1, T2, and FLAIR modalities improves classification accuracy.

All images underwent standardised preprocessing^45^, including: (1) co-registration in MNI space, and (2) skull-stripping of co-registered modalities.

### SSL model architecture

BrainFound adopts the standard ViT-Large backbone from DINOv2^23^. We use the original ViT-Large/14 configuration, consisting of a 14 × 14 patch size, an embedding dimension of 1024, 24 transformer encoder layers, and 16 multi-head self-attention heads, consistent with the official DINOv2 implementation. All architectural components of the transformer, including positional encodings, attention blocks, and feed-forward layers, remain unchanged. Input slices are resized to a fixed resolution of 224 × 224 before being fed into the ViT-Large backbone. To handle volumetric MRI data, each scan is processed as a sequence of 2D axial slices, with optional multi-contrast channels stacked along the input dimension, enabling the model to leverage slice-wise anatomical information while remaining fully compatible with the 2D DINOv2 backbone. Detailed architecture of DINOv2 is provided in Supplementary Fig. 2.

Each 3D MRI volume is represented as

1 $$V\in {{\mathbb{R}}}^{H\times W\times D\times C}$$

where *H* and *W* are the height and width of the axial slices, *D* is the depth (number of slices), and *C* is the number of channels per slice. The volume is decomposed along the depth dimension into *D* axial slices, which are processed independently using the 2D Vision Transformer backbone.

Importantly, BrainFound does not employ explicit 3D convolutions, volumetric tokenization, or inter-slice attention mechanisms. Each slice is processed independently using a 2D Vision Transformer backbone, and volumetric information is learned implicitly through exposure to multiple slices from the same volume during training. Thus, while the model processes 2D slices, it approximates anatomical context through sequential slice exposure during training.

The number of slices per volume used for training is configurable via the num_slices parameter. If num_slices ≤ *D*, slices are evenly sampled along the depth dimension; otherwise, all *D* slices are used.

For multimodal MRI scans, corresponding T1-, T2-, and FLAIR-weighted slices are stacked along the channel dimension to form a multichannel 2D input tensor.

2 $$I\in {{\mathbb{R}}}^{H\times W\times m}$$

where *m* denotes the number of MRI modalities. Single-modality scans are duplicated across three channels to satisfy the input requirement. This design allows BrainFound to process both single- and multi-contrast scans while preserving the structural information of each modality.

BrainFound follows the standard DINOv2 self-supervised learning procedure. Pretrained DINOv2 weights provide initialisation; no modifications are made to the loss function or optimisation strategy. Each slice is augmented using multi-scale cropping (global crops 96 × 96, local crops 64 × 64) and standard augmentations including colour jitter, random crop/resize, and Gaussian blur.

**Fig. 7:**
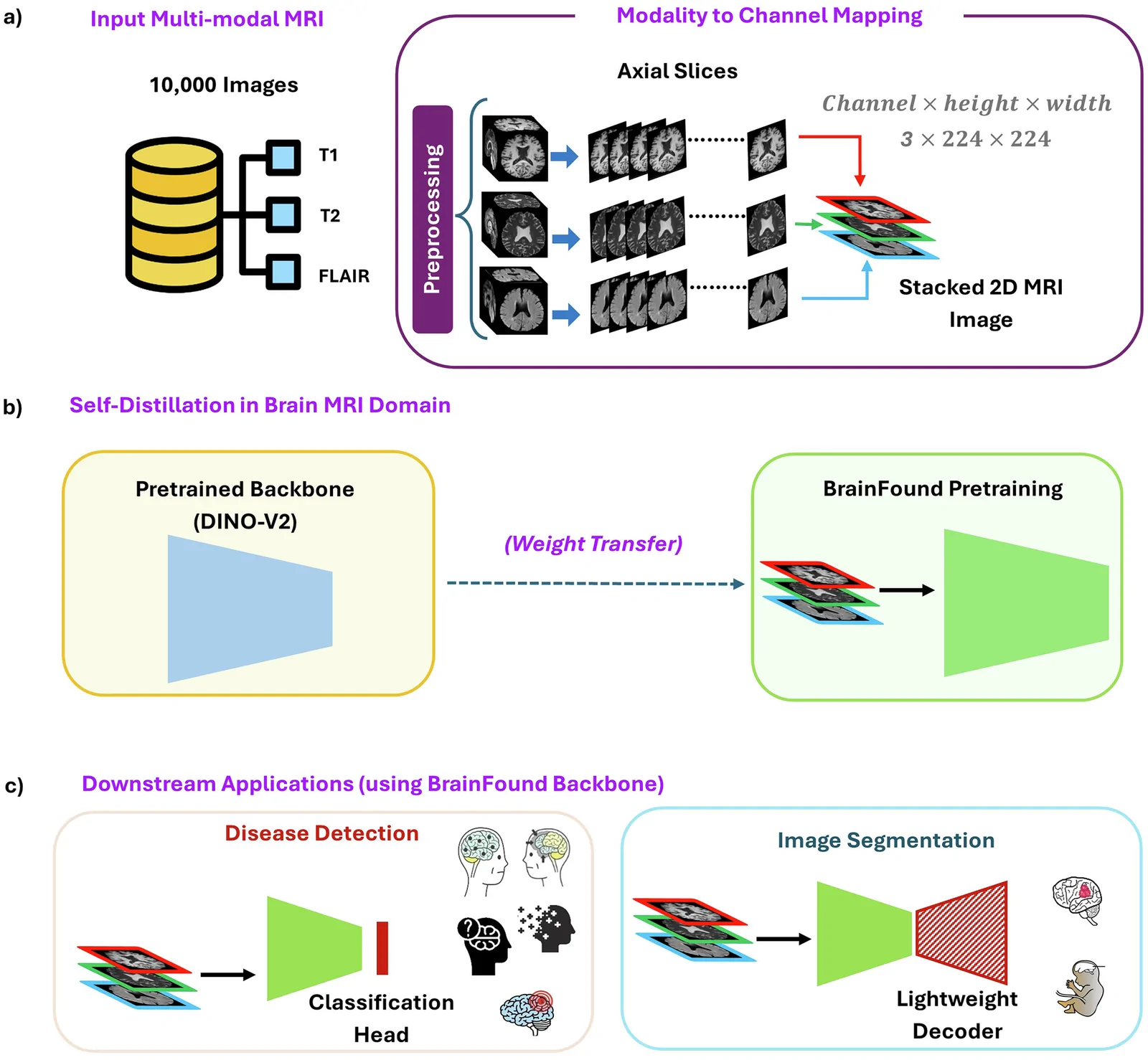
Schematic architecture of BrainFound. Schematic architecture of BrainFound. **a** Multi-contrast MRI volumes (T1, T2, FLAIR) collected from 12 public datasets ( ~ 10,000 scans) undergo preprocessing and are converted into stacked axial slice representations (3 × 224 × 224). **b** A pretrained DINOv2 backbone provides initial weights to BrainFound, which is then adapted through domain-specific self-distillation on stacked MRI slices. **c** Downstream applications: BrainFound is adapted to disease detection and segmentation tasks using a classification head and lightweight decoder, respectively.

To adapt DINOv2 to medical imaging, we leverage transfer learning techniques^29–31,33^ and self-supervised learning on multi-contrast brain MRI. A detailed pseudocode of BrainFound is provided in Supplementary Fig. 3. Spatial alignment across modalities is preserved to maintain anatomical correspondence, enabling adaptation of learned representations to downstream tasks (Fig. 7).

### Baseline models

To evaluate BrainFound’s performance and label efficiency, we compared it against three baseline models: SL_ImageNet, SSL_DINOv2, and SSL_BrainImages. All models share the same architecture and downstream fine-tuning procedures, differing only in their pretraining strategies. SL_ImageNet uses supervised pretraining on ImageNet-21k. SSL_DINOv2 is pretrained with self-supervised learning on approximately 140 million natural images from the LVD-142M dataset^23^. SSL_BrainImages is trained from scratch using self-supervised learning exclusively on brain MRI scans (T1, T2, FLAIR), enabling the model to learn modality-specific representations.

BrainFound, in contrast, initialises from DINOv2 pretrained weights and adapts to brain MRI through slice-wise self-supervised learning. This strategy leverages generic visual features learned from natural images and refines them on domain-specific multimodal MRI data, supporting downstream tasks such as disease detection, classification, and segmentation.

**Fig. 8:**
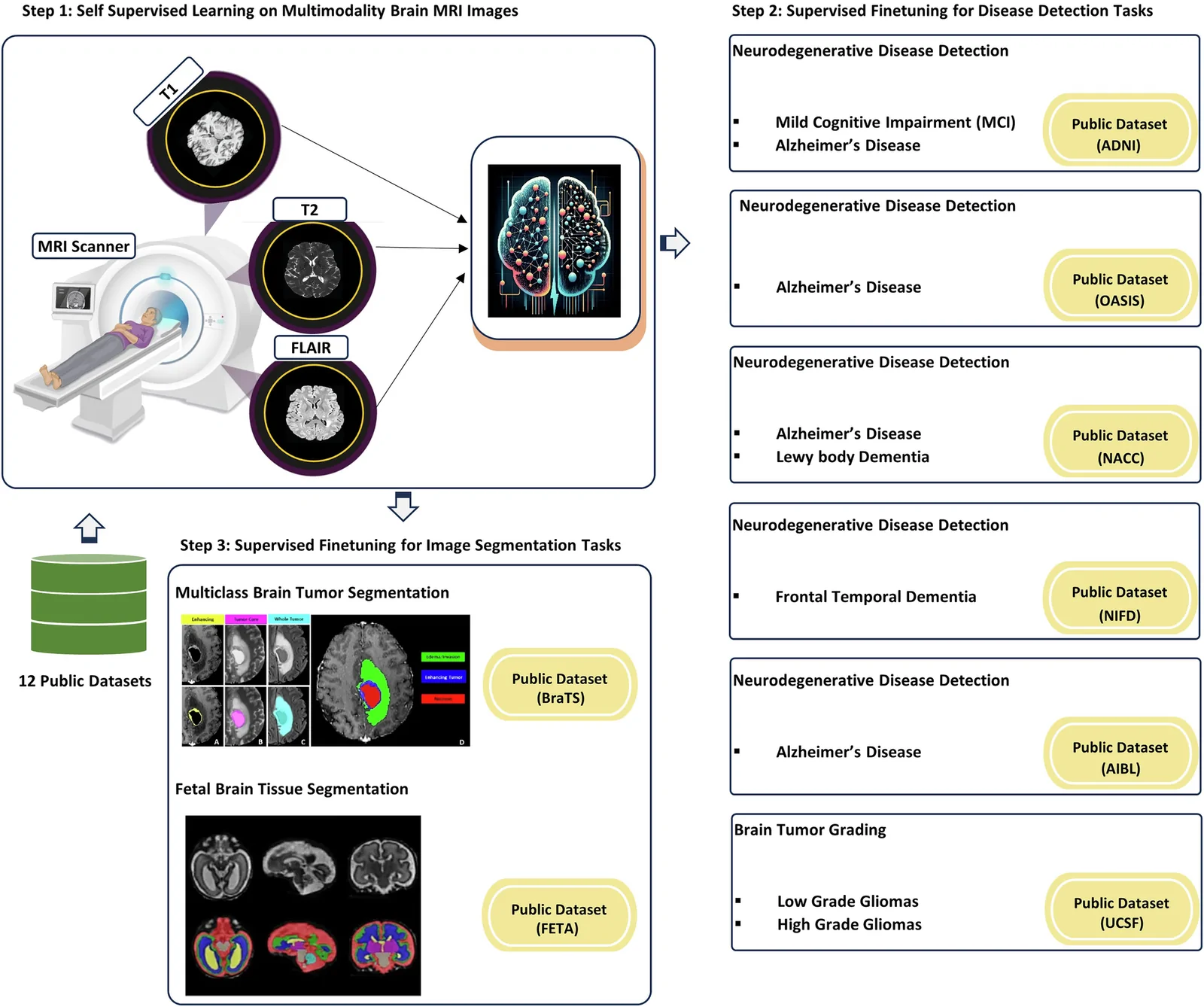
Development and application workflow of BrainFound. Overview of the development and application of BrainFound. Step one involves pretraining BrainFound using self-supervised learning on large-scale multi-scan brain MRI datasets. Steps two and three adapt the pretrained model to multiple downstream disease detection and image segmentation tasks through supervised fine-tuning, followed by both internal and external evaluation to assess generalisation across datasets.

Supplementary Fig. 1 illustrates the pretraining strategies for each model, and Fig. 8 summarises the overall development and downstream applications of BrainFound.

### Training strategy

Self-supervised learning (SSL) was performed by retraining the pretrained DINOv2 model on ~ 10,000 brain MRI volumes (Fig. 6). To adapt the 2D ViT backbone for 3D MRI data, each volumetric scan $V\in {{\mathbb{R}}}^{H\times W\times D\times C}$ was processed as a sequence of 2D axial slices, enabling spatially aware feature learning while remaining compatible with the 2D pretraining framework. ViT-Large was used as the backbone for all experiments.

To capture both global and local brain structures, a multi-scale cropping strategy was applied, generating global crops of 96 × 96 and local crops of 64 × 64 from 150 slices per volume. Each slice was processed individually through the 2D DINOv2 model with a batch size of 200 slices per iteration using mixed precision (FP16) for memory efficiency. The full DINOv2 augmentation pipeline was employed, including multi-crop, colour jitter, random crop/resize, and Gaussian blur. The average iteration time was approximately 0.5 s per batch.

Across ~ 10,000 MRI volumes ( ~ 1.5 million slices total), this setup resulted in ~ 7500 iterations per epoch, with an estimated training time of ~ 62.5 min per epoch. For 100 epochs, total SSL pretraining required roughly 104 h ( ~ 4.3 days). The model was optimised with AdamW (learning rate = 1 × 10^−4^, *β*_1_ = 0.9, *β*_2_ = 0.95, weight decay = 0.05) and trained using the DINOv2 self-distillation loss, with the teacher network updated via an exponential moving average of the student parameters. A cosine learning rate schedule with linear warmup during the first epochs improved training stability.

For downstream tasks, data augmentations included flipping, scaling, brightness and contrast adjustments, as well as RandGaussianRotate, RandZoomd, RandShiftIntensity, and RandGaussianNoise^46^. The loss function combined cross-entropy for disease detection and Dice loss with cross-entropy for segmentation. The AdamW optimiser (learning rate = 1 × 10^−4^) was used, and early stopping based on validation metrics typically concluded training around 80 epochs. Downstream training took less than 10 h with a batch size of 20 on an NVIDIA A6000 GPU with 48 GB memory.

Model performance and generalisability were assessed using a five-fold cross-validation scheme on the training data, with a strictly held-out test set (15% of volumes per dataset) reserved for final evaluation. These test volumes were never used during SSL pretraining, fine-tuning, or validation, and all reported metrics were computed exclusively on this set.

The remaining 85% of data was used for model development and cross-validation. Training data were partitioned into five stratified folds; in each fold, ~ 70% of the total was used for training and ~ 15% for validation to support model selection and early stopping. This procedure produced five distinct training-validation splits. For each fold, the best model checkpoint based on validation performance was saved. After all folds, five trained models were evaluated on the held-out test set. Metrics such as AUROC and Dice score were computed per fold, with the mean and standard deviation reported. Statistical significance was assessed using a two-sided *t*-test across folds^31^.

### Downstream tasks and evaluation scenarios

BrainFound was evaluated across multiple downstream tasks to assess its ability to capture meaningful brain representations, generalise across datasets, and perform effectively with limited annotations. These included multi-cohort neurodegenerative disease detection, brain tumour grading, external cross-dataset evaluation, few-shot learning, and anatomical and pathological brain segmentation. After SSL pretraining on multi-sequence and multimodal MRI scans, BrainFound was adapted to each downstream task using either single-modality or combined-modality inputs to reflect realistic clinical settings. Neurodegenerative disease detection employed publicly available datasets, including AD (NACC, ADNI, OASIS, and AIBL), MCI (ADNI), LBD (NACC), and FTD (NIFD). Brain tumour grading was evaluated on adult patients with histopathologically confirmed grade II-IV diffuse gliomas from the UCSF dataset^37^. Segmentation tasks included BraTS for tumour subtype lesion segmentation and FeTA for fetal brain anatomical sub-region segmentation. Dataset summaries are provided in Fig. 6, and single-versus combined-modality examples for AD detection are shown in Supplementary Fig. 4.

For rigorous evaluation, 15% of each dataset was held out as a strictly unseen test set, which was never exposed during SSL pretraining or model development. The remaining 85% of the data was used for model training and five-fold cross-validation. In each fold, four folds (70%) were used for training and one fold (15%) for validation, with model checkpoints selected based on validation performance. Final metrics, including AUROC for classification and Dice/HD95 for segmentation, were computed on the held-out test set and averaged across folds, with statistical significance assessed using a two-sided *t*-test^31^.

#### Disease detection

Performance in disease detection was assessed for both neurodegenerative and oncological conditions, testing the model’s ability to identify subtle structural and pathological patterns across cohorts and imaging modalities. Neurodegenerative classification included AD, MCI, LBD, and FTD using the relevant datasets. Tumour grading included adult patients from the UCSF dataset. AUROC was used as the primary metric, and significance was determined across five-fold cross-validation (*p* < 0.05).

#### Cross-dataset external evaluation

To evaluate robustness and generalisability, independent external datasets not used in training were employed for AD detection. This measured the model’s ability to maintain high classification accuracy across variations in scanner types, acquisition protocols, and patient populations, simulating real-world deployment scenarios.

#### Comparison of state-of-the-art self-supervised learning strategies

We systematically compared SSL strategies to justify the choice of DINOv2 as BrainFound’s backbone. Evaluated methods included SwAV^25^, SimCLR^20^, MoCo-v3^21^, MAE^22^, and DINOv2^23^. Models were trained under identical conditions on the NACC dataset (AD classification) and UCSF dataset (tumour grading), using multimodal MRI inputs (T1, T2, FLAIR). DINOv2 consistently outperformed alternative SSL approaches, supporting its selection.

#### Few-shot learning evaluation

To evaluate performance with limited annotations, few-shot experiments were conducted using 10%, 20%, 50%, and 100% of labelled training data for each task. Models were then evaluated on the full held-out test set, reflecting scenarios with scarce labels. This demonstrated the label efficiency of BrainFound and its ability to generalise from limited training examples.

#### Brain MRI segmentation

BrainFound’s ability to capture fine-grained anatomical and pathological structures was further evaluated via segmentation tasks. BraTS tumour subtype and FeTA fetal brain region segmentations were performed, processing volumetric MRI slice-wise using the 2D ViT-Large backbone with DINOv2 pretraining. Performance was quantified using Dice and HD95 scores and compared to baseline and state-of-the-art models, demonstrating strong performance across anatomical and pathological structures.

## Supplementary information

Supplementary information

## Data Availability

All datasets used in this study are publicly available and were accessed in compliance with their respective data use agreements. **BraTS**^34^: The Brain Tumour Segmentation (BraTS) dataset is available at https://www.kaggle.com/datasets/dschettler8845/brats-2021-task1. **ReMIND**^35^: Retrospective Multicenter Imaging of Neurodegeneration Dataset. Available at https://www.cancerimagingarchive.net/collection/remind/. **UPENN-GBM**^36^: University of Pennsylvania Glioblastoma Dataset. Available at https://www.cancerimagingarchive.net/collection/upenn-gbm/. **UCSF-PDGM**^37^: UCSF Paediatric Diffuse Glioma MRI dataset. Available at https://www.cancerimagingarchive.net/collection/ucsf-pdgm/. **Burdenko-GBM-Progression**^38^: Burdenko Neurosurgical Institute GBM Progression dataset. Available at https://www.cancerimagingarchive.net/collection/burdenko-gbm-progression/. **HABS**^39^: Harvard Aging Brain Study. Available at https://habs.mgh.harvard.edu/. **OASIS**^40^: A Longitudinal Study of Normal Aging and Alzheimer’s Disease. Available at http://www.oasis-brains.org. **ADNI**^41^: Alzheimer’s Disease Neuroimaging Initiative. Available at http://adni.loni.usc.edu. **NACC**^42^: National Alzheimer’s Coordinating Center. Available at https://naccdata.org. **AIBL**^43^: Australian Imaging, Biomarker & Lifestyle Flagship Study of Ageing. Available at https://aibl.csiro.au. **NIFD**^44^: Neuroimaging Initiative for Frontotemporal Dementia. Available at https://ida.loni.usc.edu/login.jsp?project=NIFD. **FeTA**^28^: Fetal Brain MRI Atlas. Available at https://feta.grand-challenge.org/feta-2022/. All data were used in accordance with relevant institutional and ethical guidelines. No personally identifiable information was accessed in this study.

## Code availability

The code used to train, fine-tune, and evaluate BrainFound is available at https://github.com/Moona-Mazher/BrainFound. The implementation is based on PyTorch. Brain MRI images were processed using the MRIPreprocessor tool (https://github.com/ReubenDo/MRIPreprocessor). DICOM files were processed using Pydicom v2.3.0. Reading and loading of 3D MRI images were performed using SimpleITK v2.2.1 and Nibabel v3.2.1. Results were analysed and visualised with Python v3.6 using NumPy v1.19.5, SciPy v1.5.4, seaborn v0.12.0, Matplotlib v3.6.1, pandas v1.5.0, Scikit-Learn v1.1.3, and Pillow v9.2.0. Additional analysis was performed using 3D-Slicer v5.4.1 and ITK-Snap v3.8.0.
