# Supplementary material for: Towards generalisable foundation models for brain MRI
Source: Npj Imaging. 2026 May 29;4:55. doi: 10.1038/s44303-026-00176-5 (PMC13639087; doi:10.1038/s44303-026-00176-5)
Supplement: Supplementary file 1 [file 44303_2026_176_MOESM1_ESM.pdf]

Supplementary Information

Supplementary Figure 1 —Comparison of pre-training strategies across models

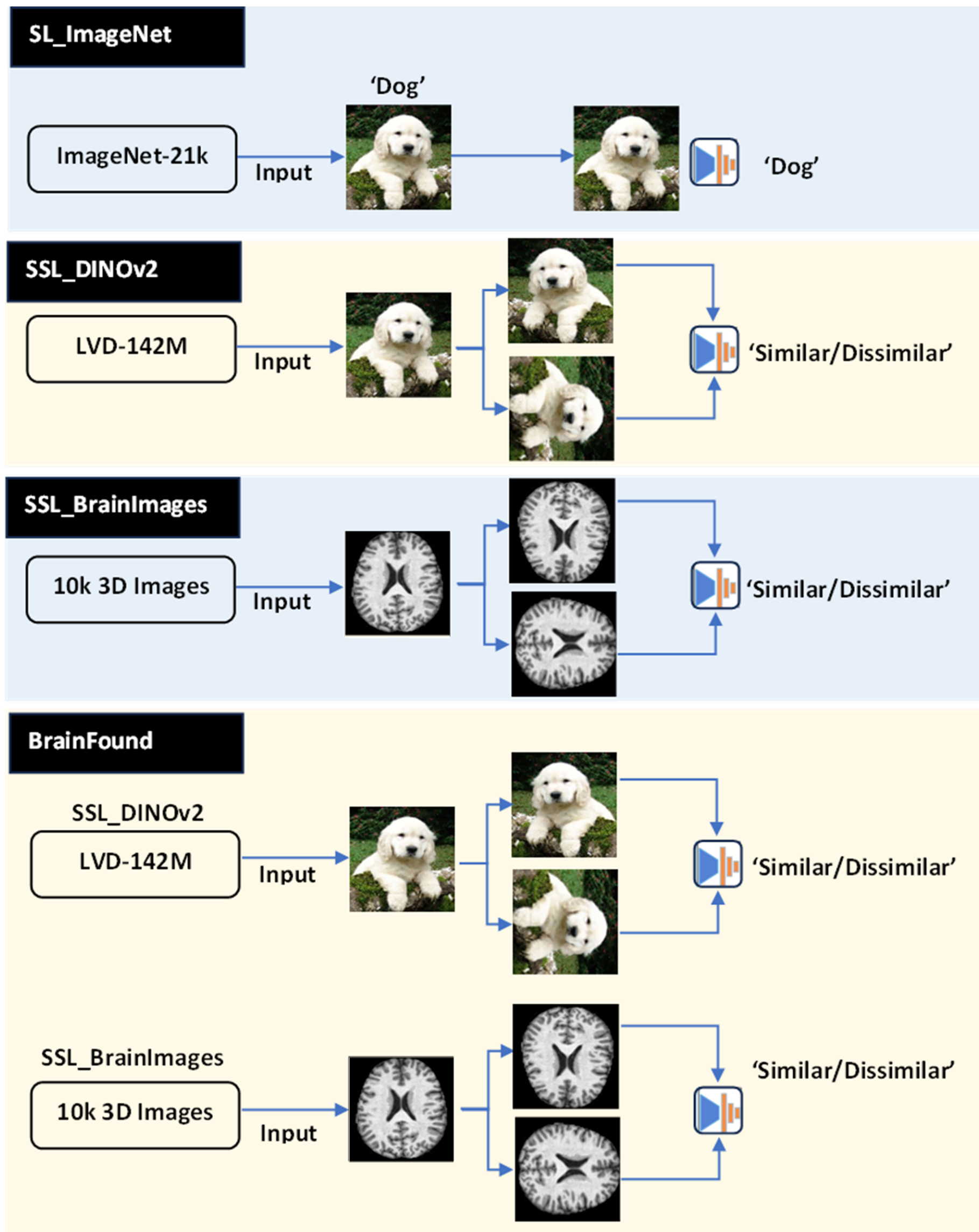

Figure 1: Overview of the training pipeline for BrainFound and baseline models. The baseline models comprised SL_ImageNet, SSL_DINOv2, and SSL_BrainImages. SL_ImageNet uses supervised learning on ImageNet-21k, which includes 14 million images with categorical labels. SSL_DINOv2 was pretrained on LVD-142M dataset. SSL_BrainImages employs self-supervised learning from scratch on brain images. BrainFound, on the other hand, utilises self-supervised learning on brain images, starting from the weights pre-trained on SSL_DINOv2. *A dog image is used to demonstrate the natural image LVD-142M dataset.

The baseline models comprise SL_ImageNet, SSL_DINOv2, and SSL_BrainImages. SL_ImageNet uses supervised learning on ImageNet-21k, which includes 14 million natural images with categorical labels. SSL_DINOv2 was pretrained on the LVD-142M dataset of natural images. SSL_BrainImages employs self-supervised learning from scratch on brain MRI images. BrainFound utilizes self-supervised learning on brain MRI images, starting from the weights pretrained on SSL_DINOv2. An image of a dog is used to illustrate the natural image LVD-142M dataset

Supplementary Figure 2 —DINOv2 Self-Supervised Learning Architecture

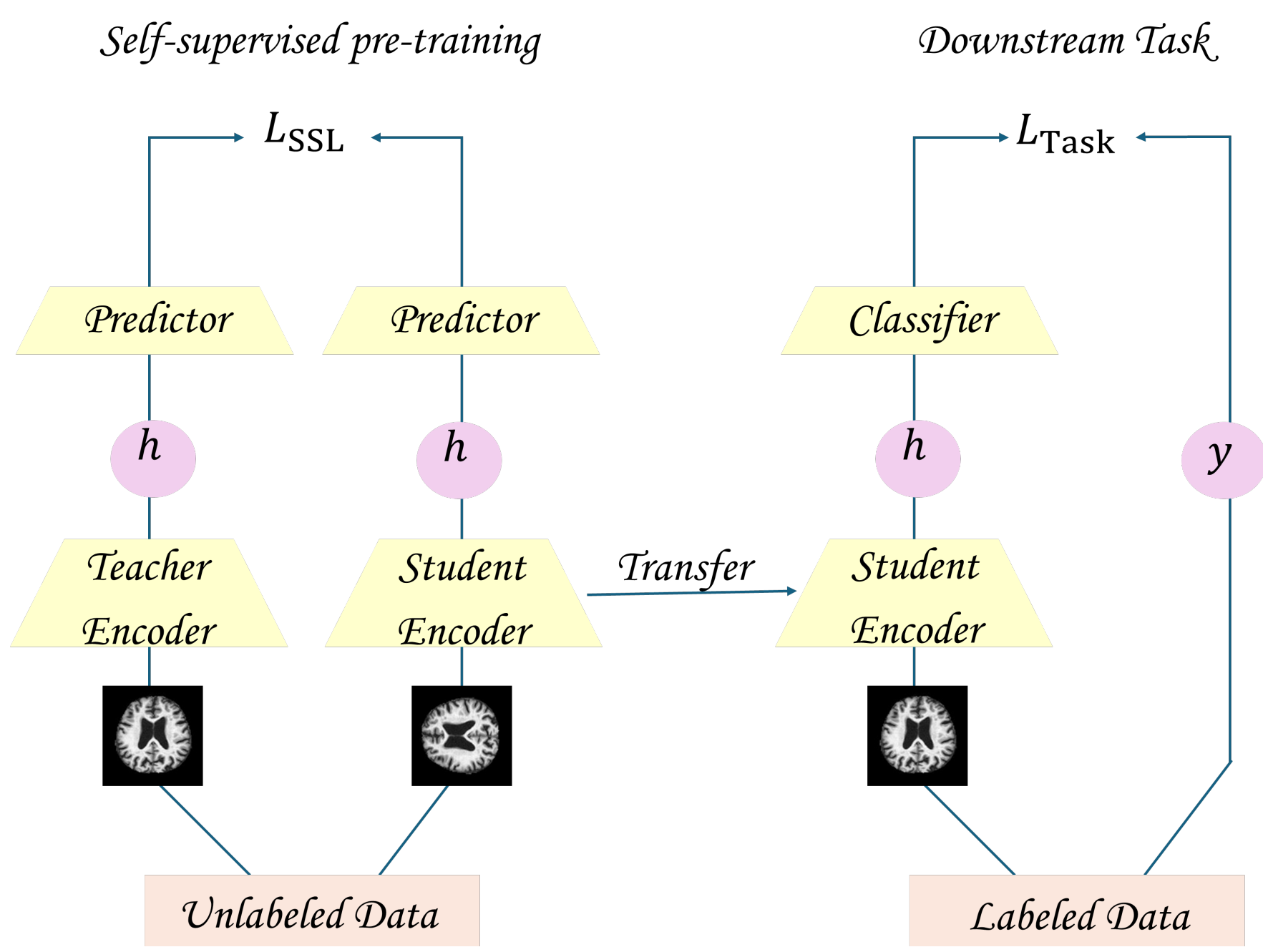

Figure 2: DINOv2 self-supervised learning architecture.

DINOv2 (DIstillation with NO labels, version 2) is a self-supervised learning framework designed to learn high-quality visual representations from large unlabeled datasets. It employs a teacher-student setup, where the student network encodes multiple augmented views of the same image and learns to match the embeddings produced by the teacher network, which is updated as an exponential moving average of the student weights. This self-distillation mechanism encourages the model to produce consistent, robust, and invariant features without requiring negative samples, unlike contrastive methods. The architecture is built on Vision Transformers, which capture hierarchical features across the

image. Once pretrained, DINOv2 embeddings can be directly transferred to downstream tasks such as classification, detection, or segmentation, providing strong generalization even with limited labeled data

Supplementary Figure 3 — BrainFound pseudocode

BrainFound Pseudocode

```
==================================

# X_list: list of MRI volumes, each of shape (1, D, H, W)
# Example: X_list = [X_t1] or X_list = [X_flair, X_t1, X_t2]
# D = total slices, H/W = height/width
# Network always expects 3 channels

# ------------------------
# User-defined number of slices
# ------------------------
user_slice_limit = USER_DEFINED_VALUE      # e.g., 50, 100, 200, etc.
D_total = X_list[0].shape[1]              # total slices in the volume
D_user = min(user_slice_limit, D_total)   # actual number of slices to process

# --------------------------------------------
# Slice loop
# --------------------------------------------
for d in range(D_user):
    # ----------------------------------------
    # Extract slice 'd' from available modalities
    # ----------------------------------------
    if len(X_list) == 1:
        # Single modality → replicate 3 times
        slice_ = squeeze(X_list[0][:, d, :, :], axis=0)  # shape: (H, W)
        Multimodality_input = stack([slice_] * 3, axis=0)  # shape: (3, H, W)
    else:
        # All three modalities → stack them
        slice_list = [squeeze(X[:, d, :, :], axis=0) for X in X_list]  # each (H, W)
        Multimodality_input = stack(slice_list, axis=0)  # shape: (3, H, W)

    # ----------------------------------------
    # Batch loop (from DataLoader)
    # ----------------------------------------
    for batch in loader:                     # batch: (B, 3, H, W)
        # Multi-modal consistent augmentations
        v1, v2 = augment(batch)            # two augmented views

        # ------------------------------------
        # Forward pass
        # ------------------------------------
        s1 = gs(v1)                         # student outputs
        s2 = gs(v2)
        t1 = gt(v1).detach()                # teacher outputs, stop gradient
        t2 = gt(v2).detach()

        # ------------------------------------
        # DINO loss
        # ------------------------------------
        loss = 0.5 * H(t1, s2) + 0.5 * H(t2, s1)
        optimizer.zero_grad()
        loss.backward()
        optimizer.step()

        # ------------------------------------
        # Teacher EMA update
        # ------------------------------------
        for ps, pt in zip(gs.parameters(), gt.parameters()):
            pt.data = l * pt.data + (1 - l) * ps.data

        # ------------------------------------
        # Center update
        # ------------------------------------
        batch_center = concat([t1, t2]).mean(dim=0)
        C = m * C + (1 - m) * batch_center

# --------------------------------------------
# DINO loss with centering
# --------------------------------------------
function H(t, s):
    s = softmax(s / τs)
    t = softmax((t - C) / τt)
    return - (t * log(s)).sum(dim=1).mean()

# Notes:
# - Supports either a single modality or all three modalities
# - Single modality is replicated to 3 channels
# - Multi-modal stacking preserves channel order
# - Slice-wise processing; user can define any number of slices
# - Augmentation applied consistently across modalities
# - Teacher network is EMA of student
# - Centering stabilizes DINO training
```

Figure 3: Pseudocode for BrainFound.

Supplementary Table 1 — Dataset distribution for SSL pretraining and downstream tasks

Table 1: Dataset distribution for SSL pretraining and downstream tasks. HABS, ReMIND, UPENN-GBM, and Burdenko-GBM-Progression were used only for SSL pretraining.

Dataset / Source	Total Images	Training / Validation	Testing
NACC [1]	1,812	1,540	272
OASIS [2]	4,134	3,514	620
HABS [3]	580	-	-
ADNI [4]	382	235	57
AIBL [5]	152	129	23
NIFD [6]	346	294	52
BraTS [7]	1,107	941	166
FeTA [8]	80	68	12
ReMIND [9]	114	-	-
UPENN-GBM [10]	1,008	-	-
UCSF-PDGM [11]	588	500	88
Burdenko-GBM-Progression [12]	360	-	-
Total	10,663	9,286	1,377

Supplementary Figure 4 — Comparison of single vs combined modalities

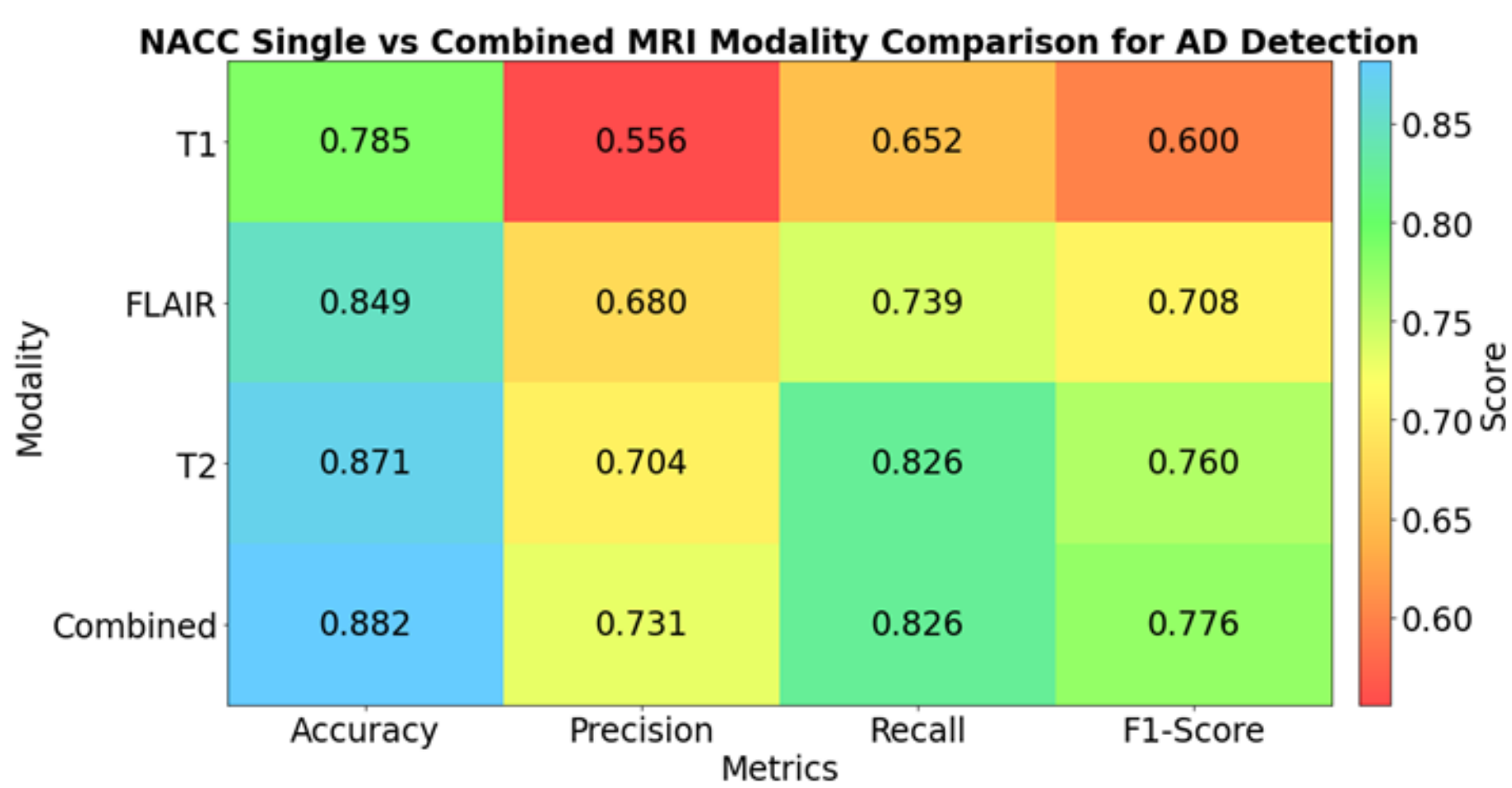

Figure 4: Comparison of single-modality (T1, T2, and FLAIR) versus combined multimodal inputs on the NACC dataset for Alzheimer's disease detection.

The figure compares Alzheimer's disease detection performance on the NACC cohort using single-modality MRI inputs (T1, T2, and FLAIR) versus a combined multimodal setting. Across all evaluation metrics—accuracy, precision, recall, and F1-score—the combined-modality configuration consistently achieves the highest or near-highest performance, indicating the benefit of integrating complementary MRI contrasts.

Among single modalities, T2-weighted images generally outperform T1 and FLAIR, particularly in accuracy and recall, suggesting stronger sensitivity to disease-related structural changes. However, no single modality achieves optimal performance across all metrics. In contrast, the combined multimodal approach yields the highest overall accuracy (0.882) and F1-score (0.776), while maintaining strong precision and recall, demonstrating a more balanced and robust classification performance.

These results highlight that multimodal fusion enables the model to capture complementary anatomical and pathological information present across different MRI sequences, leading to improved discriminative representations and more reliable disease detection compared with single-modality inputs.

Supplementary References

References

[1] Duane L Beekly, Erin M Ramos, William W Lee, Woodrow D Deitrich, Mary E Jacka, Joylee Wu, Janene L Hubbard, Thomas D Koepsell, John C Morris, Walter A Kukull, et al. The national alzheimer's coordinating center (nacc) database: the uniform data set. *Alzheimer Disease & Associated Disorders*, 21(3):249–258, 2007.

[2] Pamela J LaMontagne, Tammie LS Benzinger, John C Morris, Sarah Keefe, Russ Hornbeck, Chengjie Xiong, Elizabeth Grant, Jason Hassenstab, Krista Moulder, Andrei G Vlassenko, et al. Oasis-3: longitudinal neuroimaging, clinical, and cognitive dataset for normal aging and alzheimer disease. *medrxiv*, pages 2019–12, 2019.

[3] Alexander Dagley, Molly LaPoint, Willem Huijbers, Trey Hedden, Donald G McLaren, Jasmeer P Chatwal, Kathryn V Papp, Rebecca E Amariglio, Deborah Blacker, Dorene M Rentz, et al. Harvard aging brain study: dataset and accessibility. *Neuroimage*, 144:255–258, 2017.

[4] Ronald Carl Petersen, Paul S Aisen, Laurel A Beckett, Michael C Donohue, Anthony Collins Gamst, Danielle J Harvey, CR Jack Jr, William J Jagust, Leslie M Shaw, Arthur W Toga, et al. Alzheimer's disease neuroimaging initiative (adni) clinical characterization. *Neurology*, 74(3):201–209, 2010.

[5] Kathryn A Ellis, Ashley I Bush, David Darby, Daniela De Fazio, Jonathan Foster, Peter Hudson, Nicola T Lautenschlager, Nat Lenzo, Ralph N Martins, Paul Maruff, et al. The australian imaging, biomarkers and lifestyle

(aibl) study of aging: methodology and baseline characteristics of 1112 individuals recruited for a longitudinal study of alzheimer's disease. *International psychogeriatrics*, 21(4):672–687, 2009.

[6] Jonathan D Rohrer and Howard J Rosen. Neuroimaging in frontotemporal dementia. *International Review of Psychiatry*, 25(2):221–229, 2013.

[7] Ujjwal Baid, Satyam Ghodasara, Suyash Mohan, Michel Bilello, Evan Calabrese, Errol Colak, Keyvan Farahani, Jayashree Kalpathy-Cramer, Felipe C Kitamura, Sarthak Pati, et al. The rsna-asnr-miccai brats 2021 benchmark on brain tumor segmentation and radiogenomic classification. *arXiv preprint arXiv:2107.02314*, 2021.

[8] Kelly Payette, Priscille de Dumast, Andras Jakab, Meritxell Bach Cuadra, Lana Vasung, Roxane Licandro, Bjoern Menze, and Hongwei Li. Fetal brain tissue annotation and segmentation challenge. In *24th International Conference on Medical Image Computing and Computer Assisted Intervention (MICCAI 2021)*, page 15. Zenodo, 2021.

[9] Parikshit Juvekar, Reuben Dorent, Fryderyk Kögl, Erickson Torio, Colton Barr, Laura Rigolo, Colin Galvin, Nick Jowkar, Anees Kazi, Nazim Haouchine, et al. Remind: The brain resection multimodal imaging database. *Scientific Data*, 11(1):494, 2024.

[10] Spyridon Bakas, Chiharu Sako, Hamed Akbari, Michel Bilello, Aristeidis Sotiras, Gaurav Shukla, Jeffrey D Rudie, Natali Flores Santamaría, Anahita Fathi Kazerooni, Sarthak Pati, et al. The university of pennsylvania glioblastoma (upenn-gbm) cohort: advanced mri, clinical, genomics, & radiomics. *Scientific data*, 9(1):453, 2022.

[11] Evan Calabrese, Javier E Villanueva-Meyer, Jeffrey D Rudie, Andreas M Rauschecker, Ujjwal Baid, Spyridon Bakas, Soonmee Cha, John T Mongan, and Christopher P Hess. The university of california san francisco preoperative diffuse glioma mri dataset. *Radiology: Artificial Intelligence*, 4(6):e220058, 2022.

[12] SV Zolotova, AV Golanov, IN Pronin, AV Dalechina, AA Nikolaeva, AS Belyashova, DY Usachev, EA Kondrateva, PV Druzhinina, BN Shirokikh, et al. Burdenko's glioblastoma progression dataset (burdenko-gbm-progression)(version 1)[data set]. *The Cancer Imaging Archive*, 2023.
